# Editorial: Foodborne bacterial pathogens under the One Health perspective - antimicrobial resistance, epidemiology, virulence, and zoonotic impact

**DOI:** 10.3389/fcimb.2024.1379188

**Published:** 2024-02-12

**Authors:** Laura Maria Andrade de Oliveira, Rachel Leite Ribeiro, Erika Ganda

**Affiliations:** ^1^ Instituto de Microbiologia Paulo de Góes, Departamento de Microbiologia Médica, Universidade Federal do Rio de Janeiro, Rio de Janeiro, RJ, Brazil; ^2^ Faculdade de Medicina, Departamento de Patologia, Universidade Federal Fluminense, Niterói, RJ, Brazil; ^3^ Department of Animal Science, Huck Institutes of the Life Sciences, The Pennsylvania State University, University Park, PA, United States; ^4^ The One Health Microbiome Center, The Pennsylvania State University, University Park, PA, United States

**Keywords:** One Health, foodborne pathogens, antimicrobial resistance, epidemiology, virulence, zoonotic impact

Foodborne diseases threaten global public health as they affect human and animal health, the environment, and the food production chain. Currently, most emerging infectious diseases in humans originate from animals (Van Boeckel et al., 2015). According to the World Health Organization, nearly 600 million people (1 in 10 people worldwide) develop an illness due to the ingestion of contaminated food. Approximately 420,000 people die every year, leading to a loss of 33 million healthy life years (DALYs), with the highest burden in low- and middle-income countries (LMIC). The most affected population group is children 5 years of age, which accounts for 40% of the foodborne disease burden, with 125,000 deaths every year (World Health Organization (WHO, 2022). Additionally, foodborne diseases affect the global economy with an estimated productivity loss in LMIC of 95.2 billion per year and an annual cost of US$ 15 billion for treatment (Jaffee et al., 2019).

In this Research Topic, we navigate the intricate world of foodborne bacterial pathogens through the One Health lens, integrating human, animal, and environmental health dimensions. We highlight the most recent studies addressing aspects of molecular epidemiology, antimicrobial resistance, virulence, modes of transmission (food-to-humans, animal-to-humans, environment-to-humans), and zoonotic impact of classic and sporadic bacterial pathogens involved in foodborne diseases and zoonotic spillover events.

Zautner et al. characterized whole genomes of two multidrug-resistant *Arcobacter butzleri* isolates, an emerging diarrheagenic pathogen associated with poultry and water reservoirs. The authors evaluate antimicrobial resistance determinants, virulence potential, and genetic relationships to uncover the molecular epidemiology of *A. butzleri* causing arcobacteriosis in Ghana and inform treatment options.

Santos et al. evaluated *Campylobacter jejuni* strains to determine the duration necessary for these bacteria to fully acquire a viable but not culturable (VBNC) state. This state allows bacteria to persist in a viable yet non-culturable form, which in many cases makes it resistant to various control measures. The study focuses on the morphological changes associated with this adaptation, assessing their role in the bacteria’s adaptive and invasive capabilities. Additionally, a comparative metabolomic analysis was performed to better understand the pathogenic potential of VBNC states and their implications for public health risks. The adaptability of these pathogens highlights a concealed threat to food safety, persisting as a latent hazard despite standard control measures.

Katz et al. investigated the genetic diversity, antimicrobial resistance determinants, and virulence potential of *Campylobacter* spp. isolated from patients with acute gastrointestinal disease in Santiago de Chile and provided important insights into the molecular epidemiology of this emerging foodborne pathogen in the region.

Chopjitt et al. genomically characterized the *mcr*-mediated colistin resistance in *Escherichia coli* strains isolated from retail meat samples and evaluated the transferability of *mcr* genes by conjugation. The authors showed the *mcr* gene is localized in the bacterial chromosome and highlighted the potential of retail meat products as critical reservoirs of colistin-resistant *E. coli*.

Together, these studies underscore the complex interplay of antimicrobial resistance, epidemiology, and virulence in foodborne pathogens, reinforcing the critical need for integrated One Health strategies in addressing these global public health challenges. This Research Topic underscores the need for continuous surveillance, innovative and data-driven treatment approaches, and global cooperation to mitigate the spread and impact of these pathogens. We emphasize the call for integrated efforts in tackling these complex challenges at the intersection of human, animal, and environmental health.

We are grateful to Frontiers for allowing the effort to highlight the need for a One Health perspective when investigating Foodborne Bacterial Pathogens in this Research Topic. We hope that future studies break down siloes and take advantage of perspectives from diverse areas when investigating foodborne disease.

## Author contributions

LO: Conceptualization, Writing – original draft, Writing – review & editing. RR: Conceptualization, Writing – original draft, Writing – review & editing. EG: Conceptualization, Writing – original draft, Writing – review & editing.
